# The Vasodilatory Response to CGRP of the Anterior and Posterior Cerebral Circulation in Migraine

**DOI:** 10.3389/fneur.2022.854134

**Published:** 2022-05-19

**Authors:** Darja Visočnik, Marjan Zaletel, Bojana Žvan, Matija Zupan

**Affiliations:** Department of Neurology, University Medical Center Ljubljana, University of Ljubljana, Ljubljana, Slovenia

**Keywords:** migraine, aura, headache, CGRP, vasodilatory response

## Abstract

**Introduction:**

Migraine aura can be associated with headache or it may occur without one, which suggests an independent mechanism for the aura and for migraine headache. The role of CGRP in migraine headache is well established, but the connection between CGRP and the aura is still lacking an explanation. Exogenous CGRP can induce CGRP headaches and migraine auras in patients with migraine. The results of our recent study suggest differences in the vascular response to CGRP stimulation between migraine without aura and migraine with aura. Therefore, we hypothesized that the magnitude of the posterior cerebral circulation response in migraine with aura is greater than in migraine without aura and that CGRP stimulation has different effects on the anterior and posterior circulation in migraine with aura and migraine without aura.

**Methods:**

By using transcranial doppler, we studied the hemodynamic effects of CGRP intravenous infusion at a rate of 1.5 mcg/min in 20 min on the mean arterial velocity in the middle cerebral artery and in the posterior cerebral artery in twenty patients with migraine and in a control group of twenty healthy subjects. The same CGRP effects on cerebral hemodynamics were analyzed separately for the group of patients with migraine with aura and the group of patients with migraine without aura. Fifteen patients with migraine (75%) had migraine without aura and 5 patients (25%) had migraine with aura.

**Results:**

We found that migraine has a significant impact on the vasodilatory response of the anterior (B = 4,249, SE = 1.023, r = 0.363, *p* < 0.001) and posterior cerebral circulation (B = 3.634, SE = 1.461, r = 0.227, *p* = 0.014). Migraine with aura was significantly associated with changes in the anterior (B = 2.558, SE = 0.880, r = 0.275, *p* = 0.005) and posterior cerebral circulation (B = 7.565, SE = 2,368, r = 0.359, *p* = 0.002), while migraine without aura was only significantly associated with changes in the anterior circulation. In addition, we established a significant impact of migraine with aura on VR PCA (B = 5.901, SE = 2,546, r = 0.291, *p* = 0.024).

**Conclusion:**

We conclude that TVR in the posterior cerebral circulation might be enhanced in MA and that aura might be a consequence of TVR enhancement.

## Introduction

Migraine aura can be associated with headache or the aura may occur in the absence of a headache. These two different clinical entities suggest independent mechanisms for the aura phase and for the migraine headache phase. According to recent knowledge, the aura is attributed to cortical spreading depression (CSD) and the headache to neurogenic inflammation (1, 2). However, we cannot attribute the pathophysiologic events solely to neurogenic inflammation. Other phenomena such as abnormal cortical excitability, autonomic system instability and neurotransmitter disturbance are considered to have a pivotal role as well. The role of CGRP in migraine headache is well established, but the connection between CGRP and the aura is still lacking an explanation. However, in some patients, the infusion of CGRP triggers a migraine aura. It was also observed that treatment with gepants reduced the aura symptoms, which indicates that CGRP antagonists, or monoclonal antibodies against CGRP, may be effective in treating migraine with aura (3). Therefore, it is important to explore the role of CGRP as a link molecule between the aura and the migraine headache.

It is known that exogenous CGRP can induce headaches and migraine-like attacks in patients with migraine. It was also reported that CGRP can induce a migraine aura, which suggests a correlation between the occurence of aura and CGRP (4, 5). Silvestini et al. found that in patients with migraine with aura, there is an impairment in the adaptive cerebral hemodynamic mechanisms in the posterior circulation. This fact could have pathogenetic implications since the association between migraine and stroke frequently regards patients with migraine with aura (6). Traditionally, the migraine aura is linked to CSD. A recent scientific concept predicted CSD as an initial event for trigeminal activation, but it does not explain the connection between CGRP and the aura (7). In our previous works, we used exogenous CGRP to provoke a hemodynamic response in the anterior and posterior cerebral circulations (8, 9). The hemodynamic responses of MCA and PCA were used because we considered them as indicators of neural activity and sensitization during migraine attacks due to the trigeminovascular reflex (TVR). In the first study, we did not explore the magnitude of the hemodynamic responses separately for migraine with aura (MA) and migraine without aura (MO), but the results of our recent study suggest differences in the vascular responses between MO and MA (9). However, it is still not clear whether MO and MA show differences in the response's magnitude in the anterior and posterior cerebral circulation. We hypothesized that the magnitude of the posterior cerebral circulation response to the CGRP is greater in MA than in MO and that CGRP stimulation has different effects on anterior and posterior circulation in MA and MO. To confirm our thesis, we compared the hemodynamic response of the middle cerebral artery (MCA) and posterior cerebral artery (PCA) to the exogenous CGRP in participants with and without migraine and analyzed the hemodynamic response to CGRP of the anterior and posterior circulations in MA and MO.

## Materials and Methods

Twenty healthy participants and twenty patients with migraine participated in our study. The control group of healthy participants included 9 females and 11 males. In the migraine group, there were 15 females and 5 males. Fifteen patients with migraine (75%) had MO and 5 patients (25%) had MA. Among the MO participants, there were 10 females (66.7%) and 5 males (33.3%). Among the MA participants, there were five females (100%) and no males. The inclusion criteria were an age of more than 18 years, normal somatic and neurological status, and the absence of hemodynamically significant atherosclerotic changes in the carotid and vertebral arteries as evaluated by color-coded duplex sonography. The exclusion criteria were anti-CGRP antibody treatment, previous cerebrovascular, endocrine, renal or liver diseases, uncontrolled hypertension, pregnancy, and breastfeeding. The intake of analgesics and antimigraine prophylactic treatment was allowed. For the migraine associated headaches patients were allowed to take their proven effective drug such as paracetamol, NSAIDs and triptans. Patients with migraine were migraine headache-free for 24 h before the experiment.

The participants were free of tobacco, coffee, tea or any other food or beverages containing caffeine for at least 12 h before the start of the measurements.

All the participants were given written explanations about the experimental procedure and were informed that they were free to withdraw from the study at any time. They all gave their written informed consent to participate in the study. The National Medical Ethics Committee of the Republic of Slovenia approved the study.

TCD sonography with 2 MHz ultrasound probes was applied to measure the mean flow velocity in MCA (vm MCA) through the left and the mean flow velocity in PCA (vm PCA) through the right temporal acoustic window. During the entire experiment, the mean blood pressure (MAP) and heart rate (HR) were continuously measured using non-invasive plethysmography. The end-tidal CO_2_ (Et-CO_2_) was measured using an infrared capnograph. All the variables were recorded on the same time scale, which enabled us to compare the signals and perform correlations between them. The experiment lasted 40 min, consisting of a 10-min baseline period, a 20-min period during which an intravenous infusion of exogenous CGRP (human αCGRP, Calbiochem, Merck4Biosciences, Darmstadt, Germany) in a 1.5 mcg/min dose was administered and a 10-min period after the end of the application of the CGRP.

We defined the average values of all the parameters (vm MCA and vm PCA, MAP, HR, and Et-CO2) during 5-min intervals. T0 represented the interval during the last part of the baseline period (5–10 min of the experiment), T1 was the 5 min interval in the first part of the CGRP infusion (15–20 min of the experiment), T2 represented the 5 min interval in the last part of the CGRP infusion (25–30 min of the experiment), and T3 was the 5 min interval in the last part of the experiment after the end of the CGRP infusion (35 to 40 min of the experiment). The intervals T0, T1, T2 and T3 were used as measuring points. The vm MCA, vm PCA and mean values of MAP, HR and Et-CO2 were calculated for each 5-min interval.

In the next step, we calculated the vasodilatory responses of the vm MCA (VR MCA), as the difference between the vm MCA at T0 and the vm MCA at T1, T2 and T3 separately. VR MCA _1_ represented the difference of the vm MCA between the vm MCA at points T1-T0, VR MCA _2_ between the vm MCA at points T2-T0, and VR MCA _3_ between the vm MCA at points T3-T0. The vasodilatory responses of vm PCA (VR PCA) 1–3 were determinate in the same way.

For statistical analysis, IBM SPSS software was used (version 21, SPSS Inc.). Paired *t*-test and Student's *t*-test were used to test the significance of the differences between dependent and independent variables, as well as the chi-square test to test the differences between the frequencies. Linear regression and logistic regression were used to test the correlations between the variables. The normality of variability distribution was tested, and all the variables had values in the Shapiro-Wilk test greater than 0.05. The results of the statistical tests were statistically significant if *p* < 0.05.

## Results

**Figure 1:**
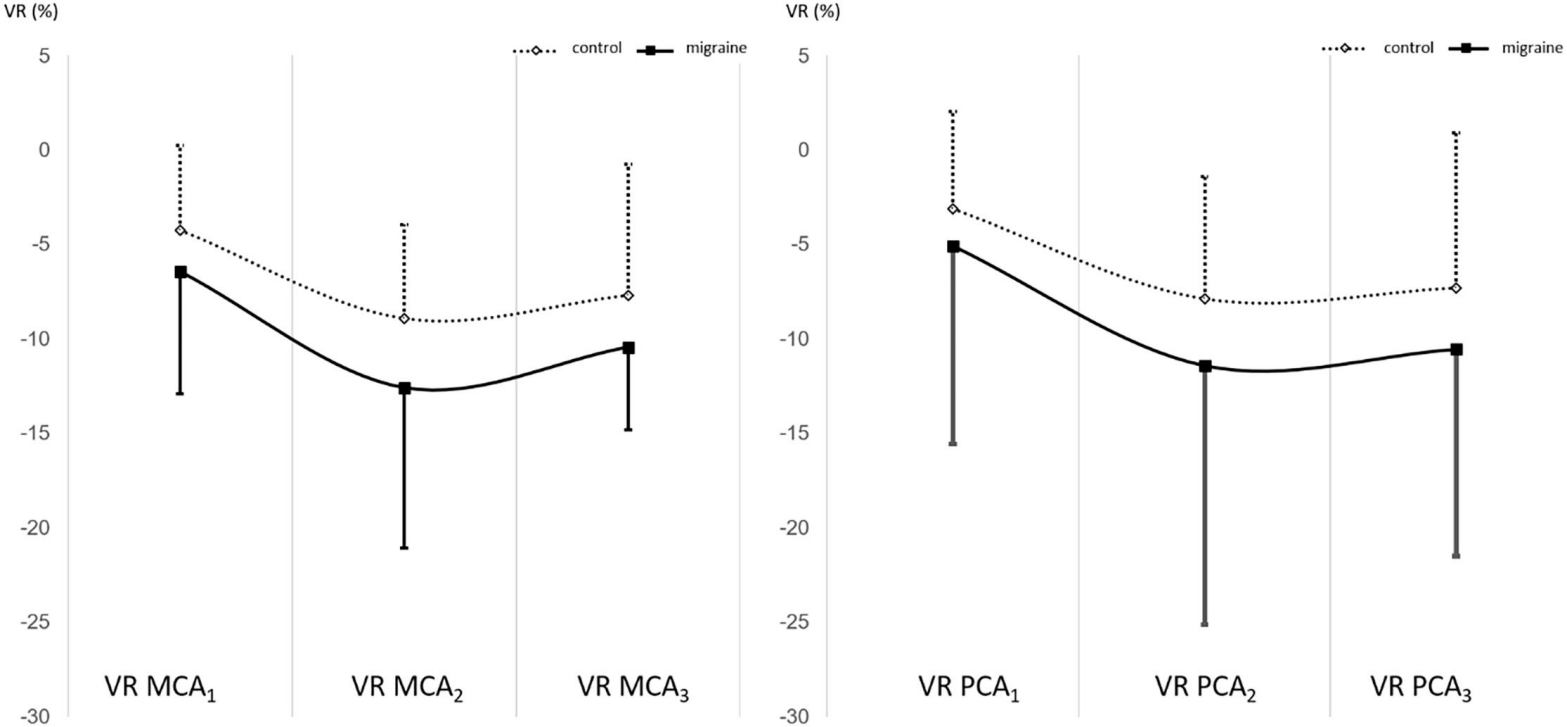
The VR MCA for migraine and non-migraine and VR PCA for migraine and non-migraine.

First, the VR MCA and VR PCA were analyzed separately for migraine and non-migraine (Figure 1). The results showed that in non-migraine subjects, an average vasodilatory response did not significantly differ between the anterior and posterior circulation (VR MCA = −6,013 ± 4.840, VR PCA = −7,892 ± 6,122, *p* = 0.200). In the migraine subjects, there were no significant differences in the average vasodilatory response between the anterior and posterior circulation either (VR MCA = −11.406 ± 8.993, VR PCA = −8.831 ± 7.987, *p* = 0.264). The *t*-test showed a significantly greater VR MCA _2_ for migraine (*p* = 0.036) compared to the non-migraine controls and no differences in the VR MCA_1_ (*p* = 0.153), VR MCA _3_ (*p* = 0,211), VR PCA_1_ (*p* = 0.315), VR PCA_2_ (*p* = 0.157) or VR PCA_3_ (*p* = 0.257) between migraine and non-migraine participants. To test the association between migraine and the VR MCA and VR PCA, we used linear regression, which showed a significant association between migraine and the VR MCA (B = 4,249, SE = 1.023, r = 0.363, *p* < 0.001) and between migraine and the VR PCA (B = 3.634, SE = 1.461, r = 0.227, *p* = 0.014).

**Figure 2:**
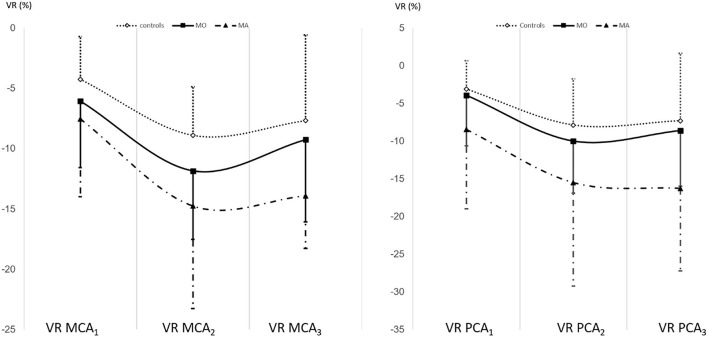
The VR MCA and VR PCA in MO and MA compared to the control group separately.

In the next step, we compared the VR MCA and VR PCA for the MO and MA group separately to the control group (Figure 2, Table 1). The linear regression showed a borderline significant association between MO and VR MCA (B = 2.112, SE = 1.154, r = 0.177, *p* = 0.070) and no relationship between MO and VR PCA (B = 1.427, SE = 1.395, r = 0.100, *p* = 0.309). Analysis of the VR MCA and VR PCA of MA group showed a significant relationship between the MA and VR MCA (B = 2.558, SE = 0.880, r = 0.275, *p* = 0.005) and between the MA and VR PCA (B = 7.565, SE = 2,368, r = 0.359, *p* = 0.002).

**Table 1:** The VR MCA and VR PCA for MO, MA and control group.

	**Anterior/posterior circulation**	**Controls**	**MO**	**MA**
VR_1_	vmMCA (cm/s)	−4.2 ± 3.6	−6.1 ± 5.5	−7.5 ± 6.4
	vmPCA (cm/s)	−3.1 ± 3.7	−3.9 ± 6.7	−8.4 ± 10.5
VR_2_	vmMCA (cm/s)	−8.9 ± 4.0	−11.8 ± 5.7	−14.8 ± 8.5
	vmPCA (cm/s)	−7.9 ± 6.1	−10.0 ± 6.9	−15.5 ± 13.7
VR_3_	vmMCA (cm/s)	−7.6 ± 7,1	−9.3 ± 6.8	−13.9 ± 4.4
	vmPCA (cm/s)	−7.3 ± 9.0	−8.6 ± 7.4	−16.3 ± 10.9

In addition, we analyzed the impact of MA on the VR MCA and VR PCA in the migraine group. We did not find any significant influence of the MA on the VR MCA (B = 3.010, SE = 1.947, r = 0.199 *p* = 0.128), while we established a significant impact of the MA on the VR PCA (B = 5.901, SE = 2,546, r = 0.291, *p* = 0.024).

## Discussion

In the present study, we observed the significant impact of migraine on the VR MCA and VR PCA. In patients with migraine, we found a significant association between the MO and VR MCA. We also confirmed the association between MA and VR MCA and an association between MA and VR PCA. Our findings suggest the existence of the enhanced reactivity of cerebral circulation to CGRP in migraine. Vasodilatation due to CGRP stimulation may be caused by endothelial-independent and endothelial-dependent mechanisms. According to the human model of migraine, CGRP is a potent vasodilatator (10). The CGRP can directly stimulate the adenylate cyclase in smooth muscle cells and trigger the production of cAMP, which activates protein kinase A and induces the opening of ATP-sensitive potassium channels and consequent vasodilatation. The important molecule for endothelial-dependent vasodilatation is nitric oxide (NO) (11). Because nitroglycerine frequently induces migraine-like headaches in patients with migraine, it seems that nitric oxide (NO) plays an important role in the pathophysiology of migraine. However, an increase in NO during migraine attacks has not been proven (12). The activation of the CGRP receptor on the endothelial cells stimulates the production of NO through the phosphorylation of nitric oxide synthase (NOS) (13). We suppose that intravenous CGRP activates the trigeminal ganglion, which is free of a blood brain barrier (14). Consequently, CGRP activity in the perivascular nerves is enhanced and the VR MCA and VR PCA are induced in an NO-dependent and NO-independent way. Indeed, some authors consider that CGRP and NO can amplify each other's activity in a reciprocal fashion throughout the trigeminovascular system (TVS), acting on the peripheral neurovascular interface within the TVS (15). Therefore, our results could be interpreted as a state of sensitization of the TVS and enhanced vasodilatation due to neuronal activity.

It is known that segmental contributions to cerebral vascular resistance occur throughout the cerebral arterial circulation which can be divided into proximal and distal segments. Proximal segments including MCA and PCA contribute about half of the overall resistance and the other half of vascular resistance in cerebral circulation is contributed by downstream of pial arterioles even at the level of capillaries (16). The infusion of CGRP may provoke important vasodilation at the arterioles' level and at the proximal segment of MCA and PCA with the consequent increase in MCA and PCA mean flow velocity at the beginning. Moreover, changes in arterioles diameter and subsequently mean flow velocity in larger cerebral arteries are strongly determined by CO2 concentration, which can reflect in our results as a decrease of End tidle-CO2. This property is called vasomotor reactivity to hyper/hypocapnia and it is one of the main control mechanisms to allow constant cerebral perfusion. Accordingly, we suggest that during CGRP provocation the proximal segment of cerebral circulation is relatively dilatated while the distal segment is contristed due to action of pCO2 to maintain constant cerebral blood flow.

Altamura et al. demonstrated that anti-CGRP antagonist erenumab preserves cerebral vasomotor reactivity to pCO2 and flow-mediated dilation in migraineurs without aura (17). In the context of our results, we may infer that while a proximal segment of cerebral circulation seems to be involved in migraine pathophysiology while distal segments remain unaffected. Therefore, outbrain structures that protected the brain, are the most important in the evolution of migraine episodes.

Additional analysis of the vascular responses in MA and MO revealed an association of MA with the VR MCA and VR PCA, while the significances of MO with the VR PCA and VR MCA showed borderline values. The results could reflect an increased response of TVS to the CGRP in MA. The differences between MA and MO are a long-term issue. Aura is a principal clinical feature distinguishing between the two clinical entities. CSD is a broadly accepted neurophysiological phenomenon as an initial event before a migraine headache, which is connected to the migraine aura. Functional imaging investigations largely agree that there is an increased activity of the brain in MA compared to MO (18). The functional imaging studies also revealed various metrics of hyper-excitability in MA. Furthermore, it was shown that CSD is tightly connected to CGRP release (19). However, the possibility that CGRP is involved in CSD has not been examined in detail. It was found that endogenous CGRP was released in the cortical tissue in a calcium-dependent manner during CSD. It is possible that the CGRP release is a compensatory mechanism to lessen CSD hypoperfusion and that the migraine headache is a side effect of this compensatory effort. From this point of view, our results indicate more pronounced TVS activity induced by exogenic CGRP due to a more inherent response of TVS in MA.

Our study showed an additional association of MA with VR PCA. This finding makes sense because clinical findings located the MA feature in structures belonging to the posterior circulation such as the occipital lobe. Furthermore, migraine represents an increased risk of silent infarct-like lesions in the posterior circulation, which are more likely in MA compared to MO or controls (20). This suggests greater CGRP effects in the posterior circulation, which could be primarily due to genetic issues or secondarily due to CGRP's compensatory role. The consequences of the more intensive CGRP stimulation of posterior circulation might be due to the combination of neurogenic inflammation of the vessel's wall and consequent hypoperfusion, which are the likeliest aetiological mechanism of infarct-like lesions. Additionally, Perko et al. found lower cerebrovascular reactivity to L-arginine in PCA in migraine (21), which indicates endothelial hypofunction in posterior circulation. However, the differences between MA and MO were not studied. The vasodilatation effects of CGRP appear to occur on the external laminae of the vessel wall rather than at the endothelial level. On the other hand, cerebral vascular endothelium is the source of NO in smooth vascular cells, and is important for the vasodilatory effect of the CGRP. Therefore, the increased VR PCA in MA could be due to the hyper-responsiveness of the TVS and the endogenous release of CGRP due to the compensation of endothelium-sourced NO deficit. This supports previous findings of increased cerebrovascular CO_2_ reactivity during the interictal period in MA compared to MO (22).

Arteries such as MCA and PCA seem to be important in TVR, representing a self-perpetuating loop, which enhances sensitization of the trigeminovascular complex and other brain structures. Our study showed a correlation between MA and VR PCA. Therefore, we might conclude that TVR acts differently in migraine and non-migraine subjects and even in MO and MA. The findings of Venieri et al. suggest improved cerebral autoregulation, a phenomenon typical connected to the proximal segment of cerebral circulation, in MA patients (23). From the point of our study, the CGRP seems to be associated with proximal segment response, which might be exaggerating in MA. In MA TVR seems to be exaggerated in the posterior circulation. This could be due to genotypic or phenotypical differences between the anterior and posterior cerebral circulation. Findings in vascular neurology on the genetic variant of RNF213 c.14576G>A indicate genetic differences between the anterior and posterior cerebral circulation (24). Nevertheless, the enhancement of TVR in the posterior circulation in MA suggests an increased sensitivity to CGRP in the posterior circulation. More intensive stimulation of the posterior circulation might induce neurogenic inflammation and ischemia in the vessel wall, which is most likely the pathophysiological mechanism behind infarct-like lesions. This is supported by the fact that migraine increases the risk of silent infarct-like lesions occurring in the posterior circulation. These lesions are more likely to occur in patients with MA compared to patients without aura or healthy subjects (20). Therefore, we conclude that aura is a consequence of a CGRP induced enhancement of the TVR. Moreover, the results of our study support anti-CGRP treatment in MA.

In terms of treatment, our results support previously gathered data on anti-CGRP treatment in migraine with aura. It is hypothesized that CGRP sensitization involves CSD generator that is located in the brainstem. Moreover, CGRP might be directly involved in CSD activation. It has been demonstrated that anti-CGRP agents reduce aura frequency by acting on the trigeminal ganglion, which is rich in CGRP-expressing neurons (25, 26). This supports our idea that exogenous CGRP acts directly on trigeminal ganglion, a region which is free of the blood brain barrier. In addition, CGRP antagonism has been proven to modulate CSD *in vitro* and *in vivo* animal experiments (27). Recent clinical studies showed a decrease of monthly headache days in patients with episodic and chronic migraine with history of aura, who were treated with anti-CGRP monoclonal antibodies (28). Safety profiles in the previously mentioned trial were similar in migraine patients with MO or MA regardless of aura history and were comparable to that of the placebo. These studies indicate CGRP involvement in aura. Therefore, CGRP antagonism shows great promise in the deveolopment of novel therapeutic strategies in MA treatment, especially since it has no important side effects.

Study limitations include low number of participants, which restricts the sensitivity of our results, and the lack of data on blood levels of CGRP in the study subjects, which might be helpful for our data interpretation. Another limitaition of our study is also a non- homogenous distribution between the two genders. The controls were not sex-matched, thus the PCA/MCA changes depend mostly on the percentage of female subjects in each group.

## Conclusion

In conclusion, it seems that TVR might be enhanced in MA in the posterior cerebral circulation. Enhancement of TVR might be clinically expressed as aura and it might be linked to genetical and epigenetical differences between the anterior and posterior circulation. Further studies are needed to investigate the CGRP-induced differences between MA and MO and the impact of CGRP on posterior circulation, especially in MA.

## Data Availability Statement

The raw data supporting the conclusions of this article will be made available by the authors, without undue reservation.

## Ethics Statement

The studies involving human participants were reviewed and approved by Medical Ethics Committee of the Republic of Slovenia. The patients/participants provided their written informed consent to participate in this study.

## Author Contributions

All authors listed have made a substantial, direct, and intellectual contribution to the work and approved it for publication.

## Conflict of Interest

The authors declare that the research was conducted in the absence of any commercial or financial relationships that could be construed as a potential conflict of interest.

## Publisher's Note

All claims expressed in this article are solely those of the authors and do not necessarily represent those of their affiliated organizations, or those of the publisher, the editors and the reviewers. Any product that may be evaluated in this article, or claim that may be made by its manufacturer, is not guaranteed or endorsed by the publisher.
